# Accepting Restrictions and Compliance With Recommended Preventive Behaviors for COVID-19: A Discussion Based on the Key Approaches and Current Research on Fear Appeals

**DOI:** 10.3389/fpsyg.2021.558437

**Published:** 2021-06-07

**Authors:** H. Andaç Demirtaş-Madran

**Affiliations:** Faculty of Communication, Başkent University, Ankara, Turkey

**Keywords:** COVID-19, fear appeals, protection-motivation theory, fear-drive theory, extended parallel process model, pandemic, health behavior, health psychology

## Abstract

COVID-19 (Coronavirus disease 2019) is a novel coronavirus which was first detected in late December 2019 in the Wuhan Province of China. This novel coronavirus, caused by a zoonotic beta-coronavirus (SARS-CoV-), is described as highly infectious. The World Health Organization (WHO) named the novel coronavirus as COVID-19 on February 11, 2020, and declared it as a “pandemic.” Almost all countries have undertaken wide-scale precautions so as to prevent or limit the spread of the virus, with most having practiced some form of “lockdown” along with “social distancing,” as well as dispensed recommendations for proper hand washing, avoiding touching the face, wearing facemasks, and using disposable tissues when either coughing or sneezing. Whereas it is well known that slowing the spread of this new epidemic requires the cooperation of all citizens, some people still seem to willfully disregard the rules and guidelines, and thereby ignore the health risks posed to both themselves and to others they come into contact with. People have responded differently to lockdown rules and social distancing practices. Whilst the majority follow the rules and recommendations with great care, others are more lax or simply refuse to comply. These differences might be accounted for according to a number of factors including personal, social, cultural, mental, and economic variables. Being persuaded to comply with preventive rules, especially those concerned with health-related behaviors, also bring certain other factors into play. Fear is one of those factors, and is one of the most powerful. It is well known that fear-based appeals can be effective in inculcating health behaviors, with many theories having been developed in this area. However, both the content of the message (the level of the fear it contains) and certain personal variables can determine the persuasive power of the fear appeal. It can even have an adverse effect if not properly applied. Many theories have been developed to address the persuasive effectiveness of the fear appeal (e.g., fear-drive theory, protection-motivation theory), and this study aims to discuss these individual differences in precautionary and preventive measures for the COVID-19 pandemic within the framework of the basic assumptions of these theoretical approaches.

## Introduction

COVID-19 (Coronavirus disease 2019) is a new type of coronavirus which was first detected in late December 2019 in the Wuhan Province of China. This novel coronavirus, caused by a zoonotic beta-coronavirus (SARS-CoV-), is described as being highly infectious. It affects the lower respiratory tract and can also manifest as pneumonia. Its most common clinical symptoms are reported as fever, fatigue, myalgia, dry cough, and dyspnea (Zhong et al., 2020). Coronavirus disease 2019 is regarded as a relative of SARS (Severe Acute Respiratory Syndrome) and also MERS (Middle East Respiratory Syndrome) (Sohrabi et al., 2020). The novel coronavirus outbreak was declared a Public Health Emergency of International Concern on January 30, 2020 and The World Health Organization (WHO) called for collaborative efforts worldwide in order to prevent the rapid spread of COVID-19 (World Health Organization, 2020b). The WHO named the novel coronavirus (2019-nCoV) as COVID-19 on February 11, 2020, and later declared it as a “pandemic,” meaning an epidemic on a global scale. Tedo Adhanom Ghebreyesus, the current serving President of the WHO, stated that the name COVID-19 is derived from the CO of “corona,” the VI of “virus,” and the D of “disease,” followed by the 19 of “2019” being the year of the disease’s official categorization.

The COVID-19 pandemic spread very quickly worldwide, with the virus having reached 215 countries and territories as of March 27, 2021, with 125,781,957 confirmed cases and 2,759,432 deaths attributed to the disease, according to the WHO (World Health Organization, 2020a). The economic and psychosocial consequences of this new epidemic have been wide-ranging, far-reaching, and unprecedented on a global scale; having caused not only significant death and serious health issues, but also severe economic problems, psychological pressures, and significant changes to daily life affecting human life worldwide. Negative psychological and economic consequences have also became another epidemic emerging alongside and as a result of the COVID-19 pandemic. The new pandemic and related economic recession has been found to be correlated with a 10-60% increase in deaths of despair in the United States alone (Sockin, 2021). The COVID-19 pandemic, as a global crisis, has resulted in disruptions to both the supply and demand of various commodities in the world economy (Chudik et al., 2020). Unemployment rates have increased, and the International Monetary Fund (IMF) described the 2020 global economy as having shrunk to an extent not seen since the Great Depression of the 1930s^1^. COVID-19 has not only affected the global population’s physical health, but has also been the cause of heightened anxiety, depressive symptoms, and stress; for example in China (Cao et al., 2020; Wang et al., 2020), Turkey (Bakioğlu et al., 2020), Israel (Tzur Bitan et al., 2020), Germany (Bendau et al., 2021), the United States (Taylor et al., 2020; Twenge and Joiner, 2020), Italy (Mazza et al., 2020), and Egypt (Arafa et al., 2021).

Numerous campaigns have been conducted in many countries to inform about the existence of COVID-19, its symptoms, means of transmission, the most at-risk groups, and the recommended precautions and preventative measures that should be taken. In order to prevent or limit the spread of the virus, governments worldwide announced new public policies such as social distancing, self-isolation, and self-quarantine (Anderson et al., 2020). People have been warned not to leave their homes (“lockdowns”), as well as being recommended how to properly wash their hands, to avoid touching their face, to use facemasks, adhere to social distancing advice, and to use only disposable tissues when coughing or sneezing. The global rates of those infected as well as numbers of deceased from COVID-19 are systematically announced through different channels of the media, with medical experts and government officials continuously warning of the serious risks presented by the virus.

Some countries have been shown to have quickly and successfully reduced infection rates, whilst some have been less responsive or effective (Hyland-Wood et al., 2021). It has been observed that not all campaigns have resulted in notable increases in effective preventive health behaviors, with individuals responding differently to lockdown rules and social distancing measures. While the majority follow these measures with great care, some largely ignore the advice and refuse to alter their behavior. These differences may be explainable through a number of determinants, including personal, social, cultural, educational, mental, and economic variables.

On the other hand, it is well known that “knowledge and attitudes toward infectious diseases” are highly associated with the level of panic emotion and, by association, with preventing the spread of communicable diseases such as COVID-19 (Zhong et al., 2020). It is therefore important to understand the public’s attitudes toward the disease, not only for helping them psychologically and physiologically, but also to help in persuading them to comply with the recommended preventive behaviors. Gerhold (2020) conducted a study with a German sample and found a negative correlation between age and the prediction of COVID-19 risk, and that females were more concerned about the new epidemic than their male counterparts. Research has shown that there has been a significant increase in the levels of depression, anxiety, general stress, and posttraumatic stress related to COVID-19 (Fitzpatrick et al., 2020; Huang and Zhao, 2020). This increase has been found to be higher for those with pre-existing depressive or anxiety disorders (Asmundson and Taylor, 2020; Bendau et al., 2021). Also, recent studies have revealed certain gender-based differences in the fear experienced in relation to the COVID-19 pandemic. Females have significantly reported higher levels of fear of COVID-19 (Humer et al., 2020; Korukcu et al., 2020; Liu et al., 2020; Qiu et al., 2020; Reznik et al., 2020; Trnka and Lorencova, 2020; Tzur Bitan et al., 2020; Koçak et al., 2021; Pak et al., 2021), depression, anxiety, and stress (Mazza et al., 2020) than males.

The rapid spread of COVID-19 has caused substantial human psychological effect worldwide, as previously mentioned (Torales et al., 2020). One of these strongest psychological effects is fear (Pakpour and Griffiths, 2020). Traumatic life events such as the emergence of new endemic diseases can cause significant fear in many people, and as such has been evaluated as a “normal” and “functional” reaction (Witte, 1992; Ahorsu et al., 2020; Harper et al., 2020; Khan et al., 2020); however, in extreme cases, this fear may lead individuals to take extreme action and even to commit suicide (Bhuiyan et al., 2020; Dsouza et al., 2020; Griffiths and Mamun, 2020; Sher, 2020).

On the other hand, fear is also known to be an effective means of persuasion, if applied properly (Witte and Allen, 2000). According to the literature on the topic of persuasion, “fear appeals” are highly associated with dealing with risk-based health behaviors, and as such have been widely used to instill attitudinal change and for different goals (e.g., coping with addiction or preventive health behaviors) (Tannenbaum et al., 2015). Recent research has established that fear is positively related to compliance with COVID-19 preventative behaviors (e.g., Bashirian et al., 2020; Cypryańska and Nezlek, 2020; Winter et al., 2020). Harper et al. (2020) found that fear was the main predictor of positive behavior changes associated with the prevention of spreading COVID-19 (e.g., guidelines on social distancing and personal hygiene). Koniak and Cwalina (2020) found that fear of COVID-19 leads to more positive and supportive attitudes toward proposed restrictions than may have initially existed.

In the circumstances of the current global outbreak, healthcare professionals and social scientists have been guiding governments in developing policies to slow down the spread of the virus and to limit its impact. At this point, it is extremely important to issue informative and persuasive messages to the population in order to ensure a higher level of virus-mitigating behaviors are adopted so as to limit the spread of the virus, and thereby to protect both their health and the health of the community at large. Persuasive messages are created and sent to society through public service announcements and similar media in many countries. Undoubtedly, the use of fear appeals is seen as one of the most effective strategies applied to increase the strength of messages in health-related persuasive communication campaigns.

In the current study, the relationship between fear and persuasion will be discussed, and then basic theoretical approaches to fear appeals will be examined in detail. In this context, complying with preventive health behaviors will be analyzed based on each of these theories’ statements. In addition, current research findings that address the relationship between fear appeals and adherence to recommended behaviors to protect against COVID-19, and thereby prevent the rampant spread of the virus, will be examined. In light of these theories and current research findings, the effects of health policies and fear-based communication campaigns that have been implemented since the beginning of the pandemic will be discussed. Finally, based on these theories, suggestions will be put forward on how to best apply messages containing fear in the most effective way in order to address the global impact of the COVID-19 pandemic.

## Fear Appeals

Fear is an adaptive emotional response to a real or a perceived physical or emotional threat that serves to stimulate the person to deal with potential risks (Gullone, 2000). Although it is considered as a negative emotion, under certain circumstances, fear can also be defined as “essential” and “functional”, as with anxiety. The optimal level of fear allows us to avoid vital or life-threatening dangers, as well as helping us adapt to the environment and to survive. For this reason, researchers working on persuasion presume “fear” to be an effective tool in replacing risk-inherent health-related attitudes (addictions, avoiding regular health checks, not adopting preventive health behaviors) with recommended attitudes and practices. In a recent study, Zettler et al. (2020) determined that the HEXACO personality domain of emotionality, which is described as excessive levels of fear, was associated with a higher level of acceptance of the restrictions aimed at slowing down the spread of COVID-19.

Fear appeals are persuasive efforts that intend to arouse fear by stressing the negative consequences (danger and/or harm) that could occur if individuals do not change their attitudes and/or practices in line with the official recommendations (Perloff, 2009; Tannenbaum et al., 2015). A fear appeal is typically structured as follows; “If you do not adopt the behavior recommended (purchasing, voting, believing, supporting, learning, stopping, etc.), you will encounter extremely negative or dangerous results.” For example, it is said that “smokers will contract chronic obstructive pulmonary disease if they do not give up the habit” or “people who do not brush their teeth regularly will soon lose their tooth if they do not develop this habit.” Considerable research has been undertaken on the persuasive effects of fear appeals (e.g., Rogers and Mewborn, 1976; Liberman and Chaiken, 1992; Eagly and Chaiken, 1993; Rogers and Prentice-Dunn, 1997; Das et al., 2003).

Although it seems a simple premise to awaken fear in people, research has shown that the same fear-arousing content does not necessarily have the same effect for all. Sometimes, a fear appeal could be less scary than the researcher’s expectation; whilst sometimes it is the opposite, with participants’ fear having become too exaggerated to take it seriously. As a result, research has shown the necessity of determining what scares who and to what extent. It is ineffectual to scare people more than necessary, just as it is to scare them too little (Morris and Swann, 1996).

Therefore, it is important to examine the variables that determine this during the current pandemic. Basic fear appeal theories provide explanations that serve this purpose. In the current study, as previously mentioned, it is aimed to make inferences in light of both the relevant theories and current research findings.

Many different theoretical approaches have been developed on fear appeals, with the following five approaches leading the literature; Health Belief Model, Fear-Drive Theory, Protection-Motivation Theory, Terror Management Theory, and Extended Parallel Process Model.

### Health Belief Model

The Health Belief Model (HBM: Hochbaum et al., 1952; Hochbaum, 1958) is one of the most commonly implemented approaches to preventive health behaviors (Rosenstock and Kirscht, 1979). It endeavors to predict health behavior in terms of certain specific belief patterns.

There are two main elements of health behavior: threat, and outcome expectations (Gehlert, 2006). The threat consists of the perceived susceptibility to health risk and also the perceived seriousness of that illness. In the case of the risks associated with being infected with COVID-19, the threat would involve believing that one was susceptible to acquiring the virus, and also that it was as serious as the health authorities portray it to be. The outcome expectations are the perceived benefits of a suggested behavior, such as using facemasks as a measure to prevent the transmission of the virus, and the perceived barriers to showing that behavior. Another element that has been added later as a separate concept is self-efficacy, which relates to confidence in one’s ability to take action (Champion and Skinner, 2008).

The combination of these factors affects the probability of the behavior happening. According to this model, fear appeals will be more persuasive if they underline a person’s perceptions about subjective susceptibility, benefits, barriers, and self-efficacy (Laranjo, 2016). The person will be more likely to adopt preventative behaviors if they perceive the health threat to be serious, that they perceive themselves to be vulnerable, and where the benefits of following the suggested behaviors outweigh the costs. If a person perceives that there is a severe threat to their health, and the perceived benefits outweigh the perceived barriers, then they will probably prefer to act in line with the recommended preventive health action.

### Fear-Drive Theory

According to Janis’ Fear-Drive Theory (Janis, 1967), fear is a driver state that motivates individuals to adopt recommendations expected to mitigate a negative state. In the case of the fear aroused in response to a message, a need for fear reduction occurs that results in an attitudinal change (Walton, 2000). According to the drive theory, the more fear that is aroused, the greater the likelihood that the fear appeal will be successful. According to this model (Janis, 1967), there is an inverted U-shaped relationship between fear and attitudinal change. In this model, it is claimed that fearful messages create a motivational urge to attitudinal change; yet, on the other hand, in the case of causing excessive fear, the expected attitudinal change will decrease.

Janis (1967) argued that the undesired high level of tension that emerges as a result of a threat motivates individuals to ignore their fears instead of changing their attitudes and adopting the proposed attitudinal change. If the recommended new attitude suggests a certain way to avoid the frightening outcome, it is possible to reduce that fear by taking the recommended route. When attitudinal change is regarded as being powerful enough to reduce or end the fear, it can be perceived as a reward, and the individual motivated to act in line with the proposed new attitude. However, if the recommended attitude is perceived as ineffective in reducing the fear, the individual will likely choose to ignore the frightening result or to reject the fear message.

To summarize, according to this theory, it is critical to find the optimal level of fear appeal at which the likelihood of attitudinal change is maximized. If the level of fear is inadequate, the corresponding fear arousal will be insufficient to initiate the expected change. On the other hand, if the resultant level of fear is considered too high, the fear appeal may cause defensive processes such as message denial or threat derogation (Manyiwa and Brennan, 2012).

Persuasion literature on fear appeals, especially those related to health-related attitudes and behavioral changes, show that when the dose of fear is misaligned, people go into defensive mode and the effect of “unrealistic optimism” appears. The term “belief in a just world” inspires this effect; where the world is perceived as just, where people live in a world in which everyone gets what they deserve. Accordingly, good things always happen to good people and bad things only happen to bad people (Lerner, 1980). Thus, the misconception that “nothing happens to me,” which is commonly observed, comes into play and forms a kind of defensive shield (Perloff, 2009). As a result, individuals who receive a fear appeal will show very little care about recommended attitudinal changes if they consider the likelihood of the fearful results to be negligible, especially when the dose of fear is significantly high. On the other hand, optimism bias can also be considered as a functional defense to cope with increased anxiety, stress and depression, especially among vulnerable groups in this process. In a meta-analysis study based on research conducted over a period of almost 70 years, it was revealed what qualifications a fearful message should contain in order to be considered persuasive (Nabi et al., 2008, 191). It was shown that such a message needs to address an important hazard through the optimal level of fear (i.e., high enough to convince the individual, but low enough not to cause undue anxiety or trigger defense mechanisms such as message denial or threat derogation) and to inform the receiver about the appropriate behavior/attitude that will enable them to avoid the risk.

The persuasiveness of fear appeals related to COVID-19 are also related to the main domains of fear. Several studies have explored these domains (e.g., Schimmenti et al., 2020; Taylor et al., 2020; Trnka and Lorencova, 2020) which include fear of contamination, fear of economic consequence, coronavirus-related xenophobia, fear of the body, fear of significant others, and fear of inaction, etc.

Fear is a subjective emotion and reactions to fear may vary from one individual to another (Mertens et al., 2020). There are apparent individual differences in tolerance for fear and risk (Lunn et al., 2020). It is extremely difficult to determine which level of fear will have what effects on which individual, to estimate the optimal dose of fear appeal, and to generalize it within the process of a global health crisis such as the current pandemic. Such messages should be supported with empathic content in order to avoid leading to perceptions of “despair” and “an inevitable end,” fed with an anxiety-reducing positive content, and emphasize that it is possible to protect oneself from the mentioned risk by taking simple measures. Perceived self-efficacy also plays a critical role in the persuasiveness of fear appeals (Peters et al., 2018; Bavel et al., 2020).

Nevertheless, debate on the persuasive power of fear appeals has continued for many years. Empirical research on fear appeals has revealed conflicting results; and whilst many studies have shown fear arousal to be persuasive (e.g., Rotfeld, 1988; LaTour and Pitts, 1989; Miller and Millar, 1998), others have concluded just the opposite (e.g., Hovland, 1959). These conflicting results may be explained by the mediating effect of “efficacy” (Manyiwa and Brennan, 2012), and Rogers’ Protection-Motivation Theory (1975) draws attention to this emphasis.

### Protection-Motivation Theory

Rogers’ Protection-Motivation Theory (PMT) (Rogers, 1975) was first developed to form a conceptual explanation of the effect of fearful messages. Rogers (1985) then went on to expand the theory with a more comprehensive version that highlighted the cognitive processes underlying attitudinal change and added extensive explanation with regards to persuasive communication.

According to this theory, individuals will choose one of the adaptive or maladaptive coping strategies after receiving the message or risk encountering significant health issues. Motivation for protection from the danger (protection motivation) occurs as a result of the perception of the danger and the corresponding evaluation of the recommended coping strategies. The greater the elicitation of protection motivation, the greater the attitudinal change.

The perception of danger represents the perception of the probability of encountering the negative experience (risk), and of its seriousness. The evaluation of coping strategies includes two elements; “response (behavioral) efficacy” and “self-efficacy.” Response efficacy is about the capacity of the new attitude/behavior suggested in the message in order to be disconnected from the danger. Self-efficacy, on the other hand, relates to the self-confidence of the individual in successfully performing the proposed attitude/behavior (Norman et al., 2005).

Perceiving health hazards and assessing the efficacy of coping strategies can lead to “adaptive behaviors” (protection motivation) or “maladaptive responses.” Maladaptive responses are behaviors that will lead to health risks for the individual. Such responses include behaviors with negative consequences (e.g., consuming too much alcohol, avoiding regular health checks for serious health issues like heart diseases and cancer). According to this theory, fear appeals can change attitudes (individuals more motivated to protect themselves from danger) under only four conditions (Rogers, 1975);

• Perceived severity/noxiousness of the danger/risk,
• Perceived likelihood of the dangerous event’s occurrence, and the perceived probability of personal damage (vulnerability/susceptibility),
• Perceived effectiveness and power of the proposed attitudinal change (measures) to prevent the risk (response efficacy), and
• Perceived self-efficacy to successfully enact the measures and practicability/applicability of the proposed new attitude.

Rogers stated that the first two conditions deal with warning individuals to change their attitudes in order to protect themselves (protection), whilst the other two are about persuading them (motivation) that they can actually achieve what is required of them. Rogers indicates that this is largely dependent on the individual’s self-belief in their own skills and perceived behavioral control perception of the severity of an epidemic plays an important role in the attitudes exhibited toward, for example, the COVID-19 pandemic. It is of the utmost importance to accurately explain the seriousness of the situation, as well as its individual, social, and even global risks to the public without any time being wasted. This perception is one of the main determinants of whether or not individuals will adopt the recommended attitudes or exhibit the advised preventive behaviors.

Consistent with this assumption, a recent study by Kuper-Smith et al. (2020) revealed that the majority of the population in both the United States, the United Kingdom, and also in Germany underestimated the possibility of being infected, did not classify themselves as being within an at-risk group, or being a carrier/transmitter of COVID-19 compared to others. They reported that individuals exhibited an “optimism bias” which led them to mitigate the probability of becoming infected themselves or infecting others. In other words, it was seen that they assumed the notion that “nothing will happen to me.” It was also observed that there was a negative correlation between the perception of “the possibility of getting infected from others” and the perception of “the possibility of participating in hygiene-related behaviors (e.g., hand washing, and social distancing).” These results could be interpreted as there being a positive correlation between the low level fear of transmission and risk-inherent social behaviors.

The second condition, in short, concerns whether or not an individual sees themself as being at risk, and is also defined as a good predictor of the recommended preventive behaviors. Wise et al. (2020) reported that with COVID-19, especially during the initial stages of the epidemic, feeling personally at risk of infection was a greater predictor of engaging in preventive behaviors such as social distancing and handwashing practices. This is closely related to the “ego-involvement” emphasized in the long-established Social Judgement Theory (Sherif and Hovland, 1961; Sherif et al., 1965). The first campaigns on AIDS (Acquired Immune Deficiency Syndrome) was acknowledged not to have worked well largely due to low levels of perceived ego involvement (Larson, 1995). At that time, it was believed that only practicing homosexuals and those from certain races were the at-risk groups, and that individuals who did not categorize themselves within these risk groups simply did not care about the messages or change their attitudes as a result. However, after the subsequent comprehension that “everyone is in the risk group,” the impact of the AIDS campaigns increased and more significant changes in attitude were seen.

The third condition answers the question, “which mitigation measures/recommended attitudes are available?” At this stage, it is extremely important to ensure that the goal of the recipient is to focus on the recommended solutions and to manage the crisis rather than focusing on their fears. If the epidemic is portrayed as a situation from which there is no escape and no remedy, individuals will panic due to high levels of anxiety and fear. Then, fear control takes over and they are unable to focus on the actual recommendations being put forward.

The final condition of “perceived self-efficacy” is concerned with the evaluation of the target person’s own ability to successfully enact the recommended measures. Encouraging people through persuasion tools such as “positive attributions” and “high expectations” can be a good solution. It is functional to state that they are believed to be able to achieve the task, and to emphasize feelings of unity so as to underline the importance of the social dimension of the spread. As reported by Harper et al. (2020), combining an acceptable level of fear with messages emphasizing personal capability can prompt safety-promoting behaviors such as personal hand hygiene and social distancing.

Using the PMT to predict preventive/protective behaviors, it may be said that those with high levels of perceived vulnerability, perceived severity of the virus, perceived effectiveness of the recommended behaviors, and perceived self-efficacy will be most likely to comply with the recommended COVID-19 protective behaviors. On the contrary, those who have a low perception of their risk, the seriousness of the virus, the effectiveness of the recommended behaviors, or their capability to following through with recommended behaviors will be less likely to engage in preventive or protective behaviors. Recently, several studies have attempted to predict the adoption of COVID-19 preventive behaviors based on PMT (e.g., Al-Hasan et al., 2020; Chong et al., 2020; Jørgensen et al., 2020; Kowalski and Black, 2021; Rui et al., 2021). Their findings have shown that efficacy beliefs predict compliance with recommended preventive behaviors in the case of COVID-19 (Chong et al., 2020; Jørgensen et al., 2020). Ezati Rad et al. (2021) found significant positive correlations between preventive behaviors for COVID-19 and perceived vulnerability, perceived severity, response efficacy, self-efficacy, and protection motivation. Kowalski and Black (2021) found that perceived severity and perceived effectiveness, and also the power of the proposed measures in preventing or reducing the risk (response efficacy) significantly predicted engagement and adherence with the recommended behaviors. Al-Hasan et al. (2020) found that higher levels of threat appraisal, coping appraisal, and intensity related to COVID-19 knowledge positively influenced social distancing adherence. Some studies have revealed that the best predictor of protective behaviors among the PMT variables is perceived severity (Anaki and Sergay, 2021; Rui et al., 2021).

### Terror Management Theory

Terror Management Theory (TMT) (Greenberg et al., 1986) asserts that mortality salience increases the potential for experiencing existential anxiety and creates a feeling of terror that negatively affects a person’s psychological wellbeing. Humans have developed two distinct buffers to cope with such feelings of terror and to feeling a sense of control over the unavoidable reality of our own mortality; and these are our cultural worldview, and self-esteem. These buffering systems alleviate existential terror by providing a sense of being a valued individual living within a meaningful world (Pyszczynski et al., 2021).

According to the model, death-related thoughts can lead individuals to either exhibit the suggested health behavior or to deny and avoid it when focused on thinking about it consciously. Proximal defenses are aroused in order to suppress such thoughts, or to push death away into the future by refusing to accept vulnerability to the risks, or motivating them to begin to act healthier in terms of their behaviors so as to ensure a longer life; namely, they lead to attempts to remove them from the consciousness. Nevertheless, in the case of thoughts about death, these are located on the fringes of our consciousness, distal defenses, which push the individual to maintain self-esteem and to cling to one’s own cultural worldview, which results in increased or decreased compliance to the proposed health behavior (Pyszczynski et al., 2021).

TMT is undeviatingly functional to understand individual responses to the current pandemic (Pyszczynski et al., 1999). According to Pyszczynski et al. (2021), the roots of the multidimensional costs (personal, social, economic, and political) of the COVID-19 pandemic are clearly based on the risk of dying from the virus.

In the COVID-19 case, the possibility of death or serious illness caused by the virus is extremely salient. Rapidly increasing mortality and intubation rates, as well as images of overburdened hospital wards make this even more evident. As such, it has been extremely challenging to manage this form of terror. Economic chaos, social isolation, human rights violations, contradictory and confusing explanations given from governments and/or scientists, as well as an infodemic spread by the mass media have also reinforced the existential anxiety and impaired our primary coping resources (FitzGerald et al., 2020).

Especially in the media, the constant informational flow regarding the virus has led to a proximal form of defense. This situation has led to an increase in problematic behaviors such as eating disorders (Ammar et al., 2020), and an increase in alcohol consumption (Furnari, 2021). Underestimating and trivializing the threat is, in a sense, another result of this defense. Dealing with positive illusions, such as not seeing one’s own age group or race at risk, evaluating the pandemic as a kind of political conspiracy, or underestimating its fatality or contagiousness, have all been exhibited. The proximal defense was also manifested by adaptive responses; compliance with recommended guidelines to avoid infection (such as maintaining social distance, hand washing, and the wearing of facemasks). Despite the ongoing information bombardment, death-related thoughts are not always at the center of consciousness. At this point, efforts to embrace the cultural worldview or to increase our self-esteem may come into play. The high correlation between political orientation, taking the virus seriously, and adhering to recommended behaviors (Funk et al., 2020) can be considered as an indicator of distal defenses.

### Extended Parallel Process Model

The Extended Parallel Process Model (EPPM: Witte, 1994) could be described as an integration of the main theoretical perspectives on fear appeals such as Fear-Drive Theory (Janis, 1967), Protection Motivation Theory (Rogers, 1975; Maddux and Rogers, 1983), and Leventhal’s Parallel Process Model (Leventhal, 1970; Leventhal et al., 1983). This theory was developed in order to illustrate the pathways that people use to appraise fear appeal messages, and the strategies they employ in response to the emotions evoked by such appeals. Although it is very similar to the Protection Motivation Theory, it contains differences in certain dimensions. Protection Motivation Theory suggests that maximum attitude change will be reached when both threat and perceived efficacy are high (Rogers and Prentice-Dunn, 1997). However, in the Extended Parallel Process Model, there are two separate types of motivation responses identified; “protection motivation response” and “defensive motivation response.” Protection motivation responses result in the acceptance of fearful messages, whilst defensive motivation responses cause rejection of the message (Timmers and van der Wijst, 2007).

According to the Extended Parallel Process Model, we face two distinct sets of appraisals when confronted with fear appeals; “threat appraisal” or “response appraisal” (Witte and Allen, 2000). In the threat appraisal, as Rogers (1975) mentioned “susceptibility,” we assess the possibility of facing a threat (e.g., “How likely am I to contract COVID-19?”) and the severity of results connected to that threat (e.g., “How harmful is the transmitted virus?”). This step is about evaluation of the effectiveness of the fear component in the fear appeal. If the likelihood and the severity are evaluated as being low, the fear appeal largely fails and the target person stops processing the message. However, if they are both high, fear leads the target to proceed to the second step, response appraisal. This step involves the evaluation of two efficacies; “response efficacy” and “self-efficacy.” Response efficacy is conceptualized as perceptions of the effectiveness of protective/preventive behaviors, whilst self-efficacy is about people’s beliefs in their own abilities to properly perform the protective/preventive behaviors (Witte, 1994).

In the case of threat appraisals and efficacy appraisals being higher than necessary, danger-control responses are triggered, and the target is motivated to engage in the recommended behaviors contained in the fear appeal message in order to protect themselves. However, if the threat appraisal is high but the efficacy appraisal is lower, fear-control responses will occur and the target will engage in maladaptive coping strategies such as denial, delay, or defensive avoidance in order to control the tensions provoked by the fear appeal.

This model also suggests that when individuals are confronted with a fearful message, one of two parallel processes (or different mechanisms) will affect their attitude; “danger control” or “fear control” (Witte, 1994). Danger control is the process that takes place when an individual believes that they can cope with the frightening result being emphasized in the message if they apply the recommended attitudinal change. For example, belief that following a recommended diet in order to cope with health problems caused by diabetes will work and succeed as desired. Fear control, on the other hand, is the process that happens when the individual focuses on how to cope with the fear that they are experiencing, instead of how they will actually deal with the frightening result they are facing.

An effective fearful message must include two main components; “danger” (a danger/risk/problem that the individual may encounter) and “detailed information about the solution” (what to do in order to deal with the problem). Such a message should first frighten the individual to a sufficient level and warn against existing dangers, and should include two different kinds of information;

• “Severity information” – E.g., Smoking can lead to a heart attack,
• “Susceptibility information” – Warning individuals about at-risk groups (e.g., All smokers are at risk of having a heart attack at an early age).

In order to achieve this, two types of information should be included; information about what they are at risk from, and the solutions they should follow.

In addition, they need to believe that these solutions will actually work and that they themselves can achieve it. In order for this to happen, two kinds of information must be included;

• “Response efficacy” – Information that emphasizes the effectiveness of the recommended attitude (e.g., Quitting smoking considerably reduces the risk of having a heart attack),
• “Self-efficacy” – Information that motivates the individual that they can cope with the problem (e.g., You can quit smoking. Millions of people have succeeded, and you can do it too).

In line with this information, the individual will enter into one of these two “parallel processes.” As previously explained, if the individual believes that they can cope with the danger, they will feel better able to face it and focus on the required solutions when executing the “danger control” process. However, if the individual believes that they are faced with serious or insurmountable danger, then they will more likely focus on their fear rather than the solution, and will enter the “fear control” process in order to try and cope with the fear rather than the danger.

To summarize, there are four basic variables in this theoretical perspective; with two related to evaluations about the threat, and two related to efficacy. In the case of the COVID-19 pandemic, the questions we need to face in terms of measuring these variables can be exemplified with the following:

Threat variables:

• Perceived severity – How serious do you think the consequences will be if you become infected with COVID-19?
• Perceived susceptibility – How possible is it that you might contract COVID-19?

Efficacy variables:

• Response efficacy – How effective do you think the recommended solutions are, such as social distancing, using a facemask, and proper handwashing at preventing COVID-19 infection?
• Self-efficacy – How confident do you feel that you could successfully follow the recommended solutions to avoid contracting the virus?

In 2006, within the Communication for Healthy Living (CHL) Project in Egypt (2003-2010), a national communication strategy was developed in order to prevent the spread of Avian Influenza (H5N1). In the campaign, the Extended Parallel Process Model was employed to persuade people to conform to certain health recommendations (Health Communication Capacity Collaborative, 2015). The study observed that whilst individuals perceived the threat posed by Avian Influenza as remaining high over time, their perceived efficacy to deal with the threat increased substantially.

The extent to which a person feels threatened by a health problem determines their motivation to act; while their confidence to efficiently decrease or prevent the threat defines the action taken.

It could be said that, in the case of a perceived threat being greater than the perceived response efficacy and self-efficacy, the probability of enacting behavioral change will likely decline. Therefore, it is extremely important to form a message that maintains balance between these two components. Message recipients must recognize that they are at risk, but at the same time understand that there are effective ways of coping with the risk and that they are capable of taking the necessary action (Witte and Allen, 2000).

Zhang (2021) found that severity and response efficacy were positively related to compliance behaviors. Increasing severity and response efficacy perceptions may be key in promoting compliance behaviors. Lithopoulos et al. (2021) conducted a test that applied EPPM in the context of the novel coronavirus disease (COVID-19) on a Canadian sample that consisted of 1,055 participants. Intentions to follow government recommendations, physical distancing, and fear control responses (i.e., negative and defensive reactions) were predicted using EPPM (perceived threat and efficacy). Consistent with the EPPM, they explored that individuals with high perceived threat and perceived efficacy scores had high intentions to comply with the recommended protective acts, physical distancing, and low fear control. Perceived efficacy was seen as the strongest predictor in their study’s analysis.

## Discussion

Individuals react to risks in many different ways; some immediately follow the recommended behaviors, whilst others opt for more harmful reactions including ignoring the reality of the risk and continuing to exhibit risk-inherent behaviors (Taylor, 2019). Although being aware of the risks of unsafe health-related behaviors, some people prefer to disregard messages designed to motivate them into changing their attitudes and choose instead to maintain their existing habits despite the warnings (e.g., Smoking Kills!) (Martin and Kamins, 2010). This personal difference is related to many variables including individual, sociopolitical, and cultural differences, A clear example of this has been seen with the COVID-19 outbreak, and which has been witnessed closely across societies on a global scale. As emphasized in detail in the current study, the use of fear appeals have effectively persuaded many people to avoid unnecessarily health risk behaviors and to practice more appropriate preventive behaviors deemed useful in dealing with global health problems such as the current pandemic.

Recent studies have revealed a relationship between fear and COVID-19 prevention behaviors (e.g., Chang et al., 2020; Harper et al., 2020; Anaki and Sergay, 2021). For example, Harper et al.’s (2020) study revealed that people who are more fearful about COVID-19 engage more with the recommended preventive health behaviors such as regular hand washing and social distancing practices. As previously noted, Zettler et al. (2020) found that the emotionality domain (i.e., having extreme levels of fear, anxiety, and reactiveness) of the HEXACO personality was positively correlated with an agreement with government-mandated restrictions.

It is well known that fear is an adaptive response in the presence of a threat. On the other hand, it is more liable to become chronic or acute rather than adaptive in extraordinary situations such as pandemics (Mertens et al., 2020). At this point, as assumed in all five of the aforementioned theories, it is vitally important to set the correct level of fear when issuing fear appeals to individuals who are perceived to be in a vulnerable emotional state. As previously mentioned, according to the Fear-Drive Theory, there is an inverse U-shaped relationship between fear and attitude change; fearful messages create a drive to attitudinal change, but, in the case of excessive fear, the expected change in attitude actually decreases. On the other hand, a low dose of fear may also cause individuals not to take the situation seriously enough, not see themselves at risk, and therefore not to heed the warnings or to obey the rules. According to terror management theory, sometimes fear appeals based on severe health risks can encourage people who gain self-esteem from risky behaviors to continue to exhibit those same errant behaviors. Then there is the “boomerang effect” (or “forbidden fruit effect”), which comes about through an increase in undesired risky behaviors, instead of following the recommended practices (Wolburg, 2006). Briefly, the necessity of adjusting the dose of fear is a well-proven concept.

It is also critical to prevent the spread of misinformation, which can lead to underestimation of the criticality of an epidemic (e.g., “nothing will happen if your immunity is good,” or “nothing will happen to individuals in the younger age group”) and blocking the transmission of incorrect messages explaining how to cope with the epidemic can prove very difficult, or even impossible in today’s multimedia online world. In the fight against COVID-19, it has been observed that many countries have placed too much emphasis on certain risk groups, and announcing that young people face a much lower level of risk has resulted in a significant reduction in their compliance with the recommended guidelines and imposed rules.

We could say that fear appeals on COVID-19 should include not only threats and risks, but also efficacy variables in light of the theories discussed in this review. The level of fear should be determined carefully in order that it does not unnecessarily lead the target audience to “fear control” rather than “danger control.” Creating effective public service announcements that include solution-oriented messages can be very persuasive if sufficient attention is paid to the level of fear aroused, and in motivating citizens that it is possible to protect themselves and society as a whole.

The significance of following the recommended measures and the other preventive behaviors must be underlined, not only as a personal health decision but also as an act of social responsibility. It should be openly declared that a pandemic requires a general community-wide approach if the mitigating actions are to achieve the desired effect. Coping with the presence and ubiquitous spread of COVID-19 requires handling as a collective aspiration rather than a set of personal goals. Researching the factors underlying the attitudes and behaviors of those who do not follow the rules is thereby also crucial.

The self-related appraisals about future events are generally optimistically biased: we consider that negative events are less likely to happen to us than to others, while the positive may be more likely. As previously mentioned, Kuper-Smith et al. (2020) argued that this bias leads people to perceive the possibility of them becoming infected, or infecting others as asymptomatic carriers, as lower than for other people. In the same study (Kuper-Smith et al., 2020), it was seen that participants from all three countries in their study (the United Kingdom, Germany, and the United States) shared optimism bias. In this process of managing a global pandemic, being aware of such biases, and exploring the underlying causes and their consequences, can help guide those responsible to form and issue persuasive messages that will further increase compliance with the recommended preventive behaviors.

On the other hand, the cultural differences observed in terms of compliance with the authorities should not be ignored when examining this kind of attitudinal change and or levels of compliance. In some countries, citizens seem to follow social isolation and lockdown rules more readily than elsewhere. For example, Anaki and Sergay’s (2021) research found that people in the United States and Europe reportedly adopted less COVID-19 related precautionary behaviors than people in Asia. Current and future research on this situation will also provide significant benefits to these and similar struggles faced by today’s national and international authorities in tackling the COVID-19 pandemic.

Being an individualistic or a collectivistic culture determines the sense of unity, interdependence, and compliance with social norms (Triandis, 2001). In individualistic cultures, the bonds between the members are considered loose; as they generally only look after themselves and their immediate family. But in the collectivist culture, people are considered interdependent, with large interconnected extended family structures, where the interests and decisions of the collective group come first based on their shared interests. Individualism tends to predominate in developed and Western countries, whilst collectivism predominates Eastern countries (Hofstede, 2011). Civics of the collectivistic eastern countries have innate cultural characteristics such as a greater tendency toward the obedience of authority, endurance, and self-discipline, and they succeed in remaining calm despite being subjected to far-reaching and often draconian restrictions in the fight against the pandemic.

Coronavirus disease 2019 protection behaviors appear closely related to the dependency/independency dimension of each culture. Interdependence cultures such as in many Asian societies afford priority to social rather than individual goals, and instill a naturally high sense of duty and communal responsibility. The importance given to social norms and the suppression of individual interests has been seen to result in greater levels of compliance with rules in such cultures (Bavel et al., 2020).

Similarly, being a tight or a loose culture also seems to be very decisive at this point. According to Gelfand (2020), it is highly related to having a “tight” or “loose” society. Tight societies are represented with strong norms and a low tolerance of deviant behavior. On the other hand, loose societies have relatively flexible social norms and a high tolerance for undesirable behaviors. Tight societies are the rule-makers, whilst loose societies are the rule-breakers. Typical “tight” societies such as Japan, Singapore, and Hong Kong have shown effective responses to COVID-19. In these countries, regularity is part of daily life, and strict, robust laws and social coordination have been shown to save lives when faced with health-related hazards such as a pandemic, especially during the early initial spread of a virus. These countries often have significant experience living with the threat or recent history of wars, natural disasters, and epidemics. Loose countries, on the other hand, like the United States, Italy and Spain are well known as more being permissive and have a softer rule-based society; and as a result face significant difficulties in managing crises such as a pandemic (Gelfand et al., 2011). Tightness-looseness indicates the extent to which social norms are pervasive, clearly defined, and reliably imposed (Gelfand et al., 2011). According to Gelfand (2018, 3), being tight or loose “not only explains the world around us but actually can predict the conflicts that will erupt–and suggests ways to avoid them.” So it could be seen as an important predictor of the ability to manage crises such as a global pandemic.

Tight cultures have generally encountered more historical epidemics, warfare, and natural disasters such as earthquakes, and the importance of applying strict rules, low tolerance for deviant behavior, and acting together in such cases has been experienced in terms of survival (Dong et al., 2021). However, in loose cultures, where freedom and individuality are highly valued when threats such as epidemics arise, it may be more difficult to become organized as a society in order to act collectively, and the restriction of freedoms is questioned far more. It is known that tight cultures such as Singapore, Japan, and China have strict social norms, and that rule violations are punished more severely in such cultures, while loose cultures such as the United States, Italy, and Brazil are more permissive (Bavel et al., 2020).

Dong et al.’s (2021) study revealed that cultural tightness is a protective factor against psychological disorders in the COVID-19 pandemic. It moderates the positive relationship found between the risk perceptions of COVID-19 with psychological disorders. Additionally, the same study showed that in these cultures people are protected from psychological disorders by the high levels of perceived protection efficacy. In another recent study, Zhang (2021) applied Hofstede’s cultural orientations (collectivism, power distance, long-term orientations, and indulgence) to examine the Extended Parallel Process Model (EPPM) (severity, susceptibility, self-efficacy, response efficacy, and compliance behavior) variables. The study’s findings showed that different fear appeal variables work differently according to certain cultural orientations. These results could be used as a functional guide to psychological prevention, and to predict compliance with protective behaviors such as those recommended in tackling the COVID-19 pandemic, and also for future probable outbreaks.

Hofstede (1980) asserted that all individuals are culturally “coded” from early childhood, and that our behaviors are generally culturally determined. Cultures also differ in their avoidance of uncertainty. A society’s tolerance for ambiguity indicates the extent to which a culture programs its members how to feel (comfortable or uncomfortable) in unexpected circumstances. It seems that avoiding cultures attempt to reduce the probability of such situations by forming strict behavioral codes, laws and rules, and an almost inbuilt disapproval of deviant beliefs and actions (Hofstede, 2011). New studies, therefore, that examine the individual differences observed in coping with COVID-19 based on culture may also prove eminently functional for our future understanding of the subject and how best to tackle it.

According to International Human Rights Law, every human has the right to the highest available standard of health. Governments have a responsibility to prevent all kinds of threats to public health, and likewise to provide medical care to those in need. It also emphasizes that, in cases of severe public health threats such as pandemics, careful attention should be given to human rights, and applications should be neither arbitrary nor discriminatory, but based on human dignity (Human Rights Watch, 2020). At the same time, it also declares that in cases of serious public health threats such as pandemics, restrictions can be justified when they are rigidly necessary, based on legal grounds and scientific data, respectful of human dignity, and proportionate to reach the goals (Amon and Wurth, 2020). In processes invoked in response to COVID-19, it has been seen that inappropriate policies have led to numerous human rights violations such as ageism, discrimination, and stigmatization (Mykhalovskiy et al., 2020), and more recently in terms of inequalities seen in access to COVID-19 vaccines. Public messages and health politics related to COVID-19 should be constructed based primarily upon a human rights approach. It is essential to combat stigma and discrimination, to respect privacy, to avoid blaming those who do not comply with the recommended measures, and fight inequities in the access to healthcare and vaccines (Mykhalovskiy et al., 2020). In addition to all of these human rights violations, there are other ethical issues to consider in the communications regarding protective measures: social distancing, the wearing of facemasks, restricted public gatherings and even private gatherings, as well as personal hygiene such as handwashing (Guttman and Lev, 2021). Handwashing and hygiene-related measures invoke certain ethical issues due to the reality of global inequity of having access to a clean and reliable water supply. Suggestions for maintaining social distance and distance-learning educational practices have brought about other examples of inequality. The suggestion to “stay at home” and to “work from home” is not readily applicable to individuals of many trades or all professions. There are also inequalities in terms of access to the necessary technological tools and infrastructure needed in terms of working or studying from home, communicating with significant others via online means, or to attending compulsory schooling through distance education.

Multidisciplinary rather than solely medical approaches are needed in order to cope with the rapid spread of a pandemic such as with COVID-19. It is essential to understand the human behaviors, attitudes, and underlying beliefs and rationales in order to be able to develop policies that are more effective on the ground. Only such an approach can contribute to the understanding of why different people have responded so diversely to the calls made by health and political authorities in the case of COVID-19 to reduce physical interaction levels and thereby the spread and impact of the disease.

In order to explain human behavior in a multidimensional context and to develop persuasive communication strategies, support is needed to be drawn from the social sciences; more specifically, from the disciplines of psychology, sociology, anthropology, and social psychology. New research is needed from the scholars of these disciplines in order to explore why people have responded so differently. Examining global health problems such as the current pandemic from the social psychological perspective could benefit not only the general public, but also politicians, educators, scientists, policymakers, and health authorities. Therefore, it is important to analyze these problems not only by conducting empirical research, but also in discussing the results according to both the traditional and contemporary theoretical perspectives. These issues should be discussed based on different theoretical approaches and from different disciplines’ perspectives in order to gather the most appropriate practical solution suggestions to cope with the COVID-19 pandemic.

## Recommendations for Awareness Campaigns

From the beginning of the COVID-19 pandemic, images and text-based messages with exaggerated and/or sensitive content with the potential to cause psychological trauma started to spread on a global scale, in newspapers, televisions, and through the ubiquitous world of social media. It was seen that pictures of individuals who had died from the virus, were hospitalized and intubated in intensive care units, and publicity shots that emphasized how the disease was rapidly progressing and had a significantly high mortality rate were shared. It has since been revealed that these messages increased the fear, anxiety, and stress levels of many people, and for some triggered seriously traumatic emotions (Dong and Zheng, 2020). Research has shown that individuals over the age of 65 years old were faced with ageist attitudes and stricter restrictions, whilst various group members identified as being “vulnerable” (e.g., healthcare providers or those already suffering from a chronic illness) were confronted with these negative outcomes to a much greater degree (Tzur Bitan et al., 2020; Trnka and Lorencova, 2020).

Although considerable evidence exists regarding the relationship between fear appeals and attitudinal change in general when it comes to disease prevention behaviors, this relationship is by no means simplistic or clear-cut. Sometimes, contrary to what is supposed in the theories, independent from the level of fear awakened, the fearful message can also be ignored, leading to defensive avoidance, or it may be so ineffective that no significant behavior change occurs (Heffner et al., 2021).

During the current pandemic, fear appeals used in health communication messages (e.g., public service announcements, posters, social media posts) have utilized graphic images and scary language to stimulate fear and to underline the negative consequences of not following the recommended behaviors (Stolow et al., 2020). However, depicting alarming and shocking scenarios can have a harmful effect, especially among those considered to be vulnerable (Lin et al., 2020; Trnka and Lorencova, 2020). Messages underlining the gravity of the pandemic could exacerbate pre-existing mental health problems such as anxiety and stress. In order to avoid such unwanted results, public messages must be designed in a way that explains appropriate ways to cope with the risks, and to increase self-efficacy in the population (Stolow et al., 2020).

The theories discussed in the current study emphasize how to deal with unwelcome negative consequences of using fear appeals. For example, in line with the EEPM’s explanations about protection motivation, public messages that support the power of the proposed measures, besides the severity of the risk, such as “The COVID-19 virus is dangerous, but do not worry, it is easy to protect you and your loved ones; wear a facemask, keep your distance from others, and wash your hands often” could be effective. It has been revealed that fear appeals are more effective when the message includes efficacy, emphasizes the severity and vulnerability of the risk, but clearly underline the applicability and functionality of the recommended measures (Tannenbaum et al., 2015). Recent studies have shown that public health messages that focus on the severity of the virus and the efficacy of the preventive behaviors are deemed to be more effective (Anaki and Sergay, 2021; Kowalski and Black, 2021; Lithopoulos et al., 2021; Rui et al., 2021).

However, some researchers oppose this method to avoid unintended consequences, which may cause the “fear control” mentioned in the fear-drive theory, and some negative socio-behavioral outcomes such as distrust in health authorities, skepticism of health messaging, and resistance to engaging in the recommended behaviors (Stolow et al., 2020), and also “news avoidance” (Tunney et al., 2021).

According to Guttman and Lev (2021), appealing to positive values such as compassion and solidarity can be an effective communication approach in cases like epidemics, where the welfare of the individual depends upon collective actions. In collective threat situations, it is useful to impart messages to the general public regarding the need to stand together and to emphasize the ethos of “Together, we can overcome this.” This strategy could prove especially efficient in the case of the COVID-19 pandemic in ensuring that individuals who are in the low-risk group comply with the restrictions and support the more vulnerable members of society by considering the general population as a community (Guttman and Lev, 2021). Many different examples of this have been witnessed; for example, the United Nations’ COVID-19 Response Creative Content Hub contains a variety of materials on prosocial acts including messages such as “Together we can overcome,” “Save people, donate to fight COVID-19,” “Follow the instructions, relax and donate,” and “Spread positive ideas, stay hopeful, stay safe” (United Nations, 2021).

Using war-type terminology (e.g., beating, fighting, enemy, weapons, victory) to motivate people to comply with the recommended COVID-19 related measures is another common communication strategy employed by political leaders (for example, former US President Trump), and also the mainstream media (Bates, 2020). Although this rhetoric seems to serve to stimulate a sense of unity against a common enemy, it is a tactic used to justify strict measures of human rights violations. On the other hand, although it seems that it aims to create solidarity, it also triggers othering, discrimination, and stigma (Venkateswaran, 2020).

There is some evidence that prosocial persuasive appeals could be more effective than fear appeals (Shen, 2011). According to Heffner et al. (2021), appeals that use prosocial language to underline the positive results of recommended behaviors can trigger positive emotions such as hope and joy, and therefore could be more effective than fear appeals in enhancing perceived efficacy.

For example, Heffner et al. (2021) used a fear appeal in their research, which included the severity of the virus and the vulnerability: “The coronavirus is coming for you. When it does, your healthcare system will be overwhelmed. Your fellow citizens will be turned away at the hospital doors. Exhausted healthcare workers will break down. Millions will die. The only way to prevent this crisis is social distancing today.” In the same research, another appeal that focused on self-efficacy and response efficacy was given to the participants with a prosocial language: “Help save our most vulnerable. Together, we can stop the coronavirus. Everyone’s actions count. Every single person can help to slow the crisis. We have the tools to solve this problem. Together, and by self-isolating, we can save millions of lives.” Their findings showed that both threat and prosocial messages were equally able to stimulate compliance with the recommended COVID-19 preventive behaviors. Therefore, considering the findings show that restrictions increase clinical mood disorders, it seems more reasonable to choose public health messages that activate positive emotions instead of fear appeals in order to increase compliance with restrictions and other preventive health behaviors. They also should be accurate, based on real data, and be transparent. They should be empathetic, and not include blaming or shaming; far from a paternalistic and authoritarian orientation, they should emphasize trust in the public, describe the rules in simple and easy-to-follow language, and highlight their practicality, ease of application, and their functionality.

Stolow et al. (2020) also proposed the use of supportive and evidence-based health communications over fear-based that explain step-by-step what can be done in order to protect themselves and society as a whole. They advised on innovative alternative strategies such as using appeals by opinion leaders and celebrities, education-based entertainment, and humor as means that could be employed as alternatives to fear appeals for the avoidance of the aforementioned unintended results. According to Guttman and Lev (2021), communication strategies should be based on the essential principles of human rights, including autonomy, equality, dignity, and privacy (Guttman and Lev, 2021). Appeals to prosocial values could be used as well as or instead of the fear appeals in light of the positive psychological perspective.

## Author Contributions

The author confirms being the sole contributor of this work and has approved it for publication.

## Conflict of Interest

The author declares that the research was conducted in the absence of any commercial or financial relationships that could be construed as a potential conflict of interest.

## Footnotes

1 https://www.bbc.com/news/business-51706225
